# A spatial-temporal linear feature learning algorithm for P300-based brain-computer interfaces

**DOI:** 10.1016/j.heliyon.2023.e15380

**Published:** 2023-04-11

**Authors:** Seyedeh Nadia Aghili, Sepideh Kilani, Rami N Khushaba, Ehsan Rouhani

**Affiliations:** a Department of Electrical and Computer Engineering, Iran University of Science and Technology, Tehran, Iran; b Australian Centre for Field Robotics, The University of Sydney, 8 Little Queen Street, Chippendale, NSW, 2008, Australia; c Department of Electrical and Computer Engineering, Isfahan University of Technology, Isfahan, 84156-83111, Iran

**Keywords:** Brain–computer interface (BCI), Event-related potential (ERP), Spatial-temporal features, Discriminative restricted Boltzmann machine (DRBM)

## Abstract

Speller brain-computer interface (BCI) systems can help neuromuscular disorders patients write their thoughts by using the electroencephalogram (EEG) signals by just focusing on the speller tasks. For practical speller-based BCI systems, the P300 event-related brain potential is measured by using the EEG signal. In this paper, we design a robust machine-learning algorithm for P300 target detection. The novel spatial-temporal linear feature learning (STLFL) algorithm is proposed to extract high-level P300 features. The STLFL method is a modified linear discriminant analysis technique focusing on the spatial-temporal aspects of information extraction. A new P300 detection structure is then proposed based on the combination of the novel STLFL feature extraction and discriminative restricted Boltzmann machine (DRBM) for the classification approach (STLFL + DRBM). The effectiveness of the proposed technique is evaluated using two state-of-the-art P300 BCI datasets. Across the two available databases, we show that in terms of average target recognition accuracy and standard deviation values, the proposed STLFL + DRBM method outperforms traditional methods by 33.5, 78.5, 93.5, and 98.5% for 1, 5, 10, and 15 repetitions, respectively, in BCI competition III datasets II and by 71.3, 100, 100, and 100% for 1, 5, 10, and 15 repetitions, respectively, in BCI competition II datasets II and by 67.5 ± 4, 84.2 ± 2.5, 93.5 ± 1, 96.3 ± 1, and 98.4 ± 0.5% for rapid serial visual presentation (RSVP) based dataset in repetitions 1–5. The method has some advantages over the existing variants including its efficiency, robustness with a small number of training samples, and a high ability to create discriminative features between classes.

## 1 Introduction

Brain-computer interface (BCI) systems represent a class of communication systems that allows people to interact with external devices by using brain signals [1,2], and are frequently used to study, map, support, improve, or restore human cognitive or sensory-motor processes. BCIs are utilized for various applications such as spelling, painting artwork, controlling a smart home, designing games, stroke rehabilitation, and furnishing internet tasks [3]. The speller systems are the most popular and widespread type of BCI applications that can help patients to write their thoughts just by focusing on the virtual keyboard through brain signals without using their hands [[4], [5], [6]]. These applications are suitable for patients who are unable to use their muscles normally, such as amyotrophic lateral sclerosis (ALS), spinal cord injury (SCI), Duchenne muscular dystrophy (DMD), and stroke [7,8]. Electroencephalogram (EEG) is often used for measuring non-invasive brain signals in BCI applications such as steady-state visually evoked potential (SSVEP) [9,10], P300-based event-related potential (ERP) [11], sensorimotor rhythm (SMR) [12], and slow cortical potential (SCP) [13]. ERP-based paradigms are so efficient in practical BCI speller systems, given their limited associations to eye fatigue problems [[14], [15], [16], [17], [18], [19], [20], [21], [22]]. ERPs are small domain potentials that are generated in response to specific events or stimuli on the EEG signal. There are different ERPs of the brain including P50, N100, N200, N300, N400, P300, and P600. The P300-based ERP signals that are associated with cognitive information processing in the brain are analyzed in the current study.

The P300 component is the positive potential in the EEG signals that occurs approximately 300 ms after task-relevant events in an “oddball” paradigm. A common paradigm based on the speller system is the row-column paradigm (RCP) that is designed by Farwell and Donchin [19], with a character matrix in which the rows and columns of it intensify randomly. If a specific character is attended, the subject focuses on the intended character that flashes while ignoring any flashes that do not include the target character. Since the flashing of the intended character is rare, it could elicit a P300 component. Following the RCP paradigm, many types of P300 speller paradigms were introduced including the single character paradigm (SCP) [23], T9 paradigm [17], checker board paradigm (CBP) [16], rapid serial visual presentation (RSVP) [14,18,22], and region-based P300 paradigm (RBP) [15]. Among them, the important kind of the P300 paradigm is the RSVP that is created by some blocks with each block including symbolic groups presented randomly to the speller's user.

In the P300-based ERP signal analysis, linear discriminant analysis (LDA) is a common approach for ERP linear subspace learning algorithms to improve signal representation [23]. The LDA method, with its simple and practical implementation, is a suitable tool for binary discriminant analysis of ERP signals. To further tackle the computational cost problems of some LDA variants, the maximum margin criterion (MMC) [24] has been proposed as a computationally low-cost variant of LDA [25]. Moreover, the conventional linear-based method such as l1-norm Lasso regularized linear model [26], Bayesian linear discriminant analysis (BLDA) [27], group-Lasso (gLasso) [28], group-sparse BLDA (gsBLDA) [29] were introduced to improve the P300 analysis performance. However, these linear methods require a lot of features and a long calibration time to be accurate [30]. Following earlier work, the stepwise LDA (SWLDA) [19], and the shrinkage LDA (SKLDA) [31], were proposed while using a smaller training sample size for learning the model. Although these methods have acceptable performance in problems with a small number of samples, since the input is in the form of a vector, the high ratio of feature dimensions to the number of samples causes ill-conditioning. Moreover, since both spatial and temporal features are efficient in the P300 signal [32], they should be included in our analysis.

In previous literature, spatial-temporal algorithms (called bilinear mapping) have been suggested to recognize the spatial and temporal projection matrices in P300-based signals [[33], [34], [35]]. Yu et al. introduced a bilinear common spatial pattern (BCSP) to accommodate both spatial and temporal information [33]. The method was a generalized common spatial pattern (CSP) by iterative optimization on spatial and temporal ERP features. Recently, Jalilpour and Hajipour proposed a new regularized CSP-based algorithm called regularized common tensor pattern (RCTP) to classify P300 signals based on RSVP speller [34]. Although their methods could reduce the noise effects and overfitting aspects, the mean accuracy of the RCTP method for all subjects didn't change significantly in comparison with the BCSP method. Zhang et al. introduced spatial-temporal discriminant analysis (STDA) for the ERP classification [35]. The superiority of their method was that the STDA required less training samples than other modified LDA (stepwise LDA, regularized LDA). However, The main limitation of the STDA is the lower performance for predicting large numbers of test data compared to recent report articles on the state-of-the-art P300 data [29,36,37]. Furthermore, the aforementioned spatial-temporal methods have some limitations, including limited interpretability and challenges with high-dimensional data. In high-dimensional datasets, where the number of input features is much larger than the number of samples, the covariance matrix can become singular, making it difficult to estimate the parameters and resulting in poor performance. Additionally, while these methods can yield good classification results, they may not provide sufficient insight into the underlying mechanisms that drive the classification, as they use a linear combination of input features. Moreover, these methods are often sensitive to outliers, which can skew the results and reduce the accuracy of the algorithm. Therefore, in the current study, we introduce novel spatial-temporal linear feature learning (STLFL) algorithm which makes a tradeoff between required low-training samples and high performance. Furthermore, the STLFL algorithm maps the raw signal to lower dimension space with higher discriminative ability compared to the literature based on LDA [24,35,38]. The use of two hyper-parameters in the cost function of the STLFL algorithm with a dimension reduction approach can help to mitigate the impact of outliers. This is achieved by decreasing the within-class distribution and increasing the between-class distribution simultaneously in lower-dimensional features, making the algorithm less sensitive to outliers.

A crucial part of the character recognition aim of the speller is choosing a fit classifier technique. In recent years, researchers found that Deep Learning (DL) models have been proven as powerful methods with a high capability of generalization to image analysis [39], and signal processing [40]. One of the DL branches is a restricted Boltzmann machine (RBM) [41] which applies in unsupervised approaches. However, some features learned by RBM are useless for classification because there is no discriminative ability in the RBM algorithms. Therefore, discriminative RBM (DRBM) was introduced to overcome this challenge [36,37,42] and demonstrates higher performance than previous methods in the character recognition approach [36,37,42]. In this paper, we apply this technique to classify extracted features by STLFL. The main innovations of the work are as follows:

• The STLFL method is proposed to extract the more discriminative P300-ERP features and could map the features to the lower dimensional space.
• The proposed STLFL achieves high robust performance using a small number of training samples.
• A new model is proposed based on the STLFL feature extraction and DRBM classifier (STLFL + DRBM) to detect the P300 and non-P300.
• The STLFL + DRBM can help to reduce overfitting and enhance the performance of the model, whether using large amounts of training data or small amounts of training data.

The effectiveness of the proposed technique is evaluated using three BCI speller datasets (two BCI competitions dataset [43,44] and an RSVP dataset [45]) and the results are compared with the previous works. The results verify that accurate classification and high information transfer rate (ITR) are obtained using the proposed new P300-based BCI scheme.

The rest of this paper is organized as follows. Section 2 details the materials used and the proposed method containing feature extraction and classification approaches. Section 3 presents the simulation results of the study. Finally, Section 4 provides a discussion of the results, including their implications and limitations. Moreover, the conclusion of the study and summarizing the main findings, along with their potential implications for future research are presented in Section 4.

## 2 Materials and method

### 2.1 Datasets and preprocessing

**Fig. 1:**
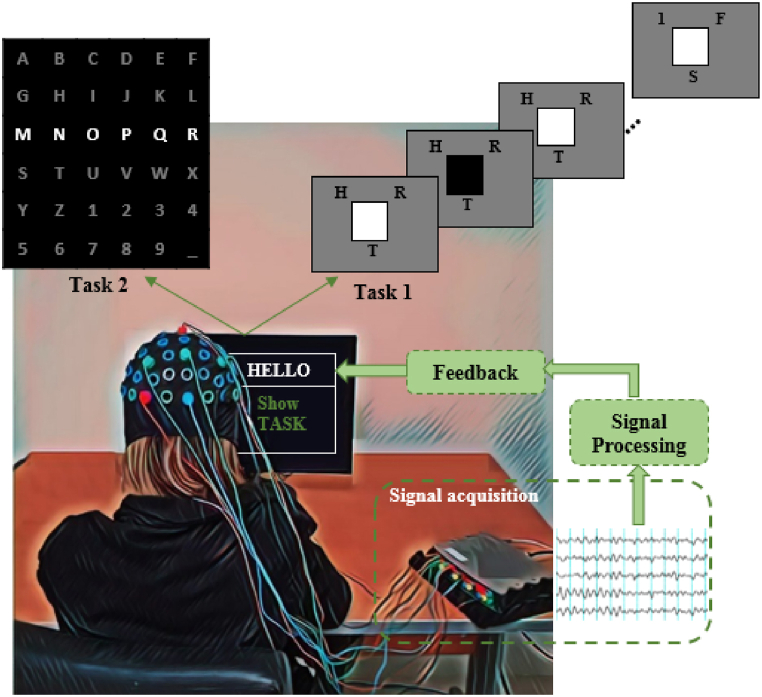
Overall schematic of the BCI speller of two task-related for two datasets (Task 1 for the hybrid dataset and Task 2 for the BCI competitions dataset). The subject sits in front of a monitor that shows the BCI speller task and the EEG signals are acquired by the EEG-recorded device and its accessories (including 10–20 EEG cap, and electrodes). Then, the collected data are processed and the character is recognized as shown in the feedback block on the monitor.

All BCI speller experiments performed in Ref. [45] and BCI competitions [43,44] were approved by the Ethics Committees of Iran's Medical Sciences and Wadsworth Center, NYS Department of Health, respectively, and their acquired datasets were used in the current study. The experiments were carried out in accordance with the relevant guidelines and regulations. All participants provided written informed consent according to the institutional guidelines for both datasets. Fig. 1 illustrates the overall schematics for both types of datasets. The subject sits in front of a monitor and focuses on the speller task. The electrodes are placed on the head and record the EEG signals by a multichannel device. The details of two speller datasets are presented in the following sub-sections:

#### 2.1.1 Hybrid RSVP-SSVEP dataset

The hybrid dataset was recorded from six subjects for the RSVP-SSVEP paradigm based on BCI speller in the literature [45]. As can be seen in Fig. 1 (Task 1), there are 27 alphabetic letters divided into nine sub-groups that contain three different alphabetic letters surrounded by a flickering square at 15 Hz, and a group (containing nine sub-group) shown randomly five times (repetitions) on the screen (see details in Ref. [45]). The target sub-group of characters is detected by RSVP stimuli and a target character on the target sub-group is identified by SSVEP stimuli. The experiment consisted of 24 offline runs, with each run requiring the subject to spell 3 characters (3 trials) and respond to 45 stimuli per trial. During the trials, the subject focused on the symbol group, which contained the target character, and silently counted the number of times the target stimulus appeared. In all repetitions of the trial, the target character was presented five times in the same symbol group and position. The duration of each P300 stimulus was 230 ms, and there was no inter-stimulus interval (ISI) between stimuli. As a result, it took a total of 10.5 s to select a character. The EEG signals were recorded by 32-channel g. Hlamp device, with a sampling rate of 512 Hz. All recording channels were connected to the right earlobe electrode as a reference channel and the electrode on the forehead is considered as ground (GND). All EEG data of 32 channels are used for P300 analysis. The current study only used the RSVP-based signal for the evaluation of the proposed algorithm. The EEG data are band-pass filtered with the cutoff frequency of 0.5–25 Hz (FIR-type). The filtered data are then segmented into 1 s epochs and decimated by the factor of 10. The preprocessed RSVP signals are then entered into the feature extraction algorithm.

#### 2.1.2 BCI competition III dataset II

This dataset was recorded from two subjects for P300 BCI speller in the literature [43]. The task was to sequentially focus attention of the subjects on the predetermined character in a 6 × 6 alphanumeric character matrix (traditional raw-column paradigm). As can be seen in Fig. 1 (Task 2), each row and column in the matrix was randomly intensified, therefore, there are 12 intensifications in which the P300 coefficient occurs in the subject brain after focusing on the intensification. To detect a character of matrix speller, each intensification repeats 15 times (repetition) so, there are 15 × 12 = 180 intensifications for each character recognition called trials in this section. Each flash duration was 100 ms and the ISI was 75 ms (see details in Ref. [43]). The objective was to identify the P300 coefficients in the rows and columns response of the brain and choose the correct characters by intersecting them.

The EEG signals were recorded by a 64-channel device with a sampling rate of 240 Hz. The data of all channels are analyzed in the preprocessing phase. The P300 signals are filtered with a third-order band-pass Butterworth filter with a cutoff frequency of 0.1–30 Hz. All P300 data are segmented into a window with a 667 ms length and the sampling rate is reduced to 40 Hz. Since 85 characters were spelled in the training phase and 100 characters in the testing speller phase, in this study, all the results are used for recognition of the 100 test characters.

#### 2.1.3 BCI competition II dataset II

This dataset includes subject C and it is similar to the BCI competition III Dataset II, with the exception that it has 42 pre-detection characters for the training phase and 32 characters for the testing speller phase [44]. However, there is an issue with the event cue in the final three characters of training data [46], causing only 39 characters to be utilized for training the classifiers of the model.

The imbalance in the numbers of P300 and non-P300 samples in the aforementioned datasets (2.1.1, 2.1.2, and 2.1.3) can lead to bias and inaccurate classification results, especially when the non-P300 samples dominate the dataset. To address this issue, we balanced the numbers of target and non-target samples by replicating the P300 samples, so that there were equal numbers of P300 and non-P300 samples [47]. This approach helped to reduce the impact of bias in the classification process, resulting in a more reliable and accurate result.

### 2.2 Architecture of the proposed model

**Fig. 2:**
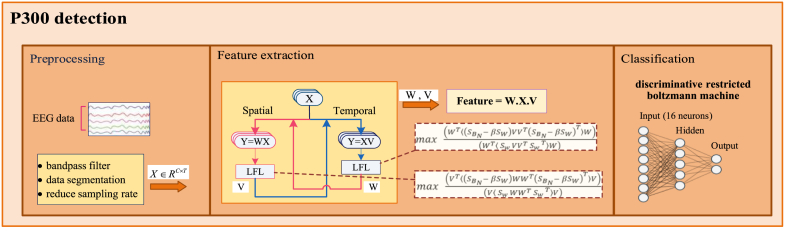
P300 detection-based character recognition: A multistage model of preprocessing, feature extraction, and classification.

The overall schematic of the proposed method is shown in Fig. 2. There are three analysis phases containing preprocessing, feature extraction, and classification. First, the EEG data are filtered with band-pass Butterworth filter (cutoff frequency: 0.5–25 Hz for the RSVP dataset and 0.1–30 Hz for both BCI competitions), segmented, decimated (see the details of preprocessing phase for two datasets in section 2.1.), and then, the novel STLFL algorithm is applied to preprocessed data (${X=R}^{C\times T}$, where *C* denotes spatial features and T indicates temporal features) to find the optimal spatial and temporal mapping weights (*W*, *V*). Mapped features by *W* and *V* have lower dimensions compared to *X* to have a more discriminative ability. Finally, a DRBM with five hidden neurons is utilized for the classification approach to recognize the P300 and non-P300 classes (section 3.1). All analyses are performed on a laptop with an i7 processor, 8 GB of RAM, and a GPU unit (NVIDIA GeForce 940MX) in MATLAB R2019b (64-bit) under Windows 10.

#### 2.2.1 Spatial-temporal linear feature learning (STLFL)

Due to the various responses of different subjects to the same P300-based paradigm, P300-based ERP requires a robust technique to be more applicable for all subjects. The Fisher linear discriminant (FLD) analysis is widely used to find a projection matrix (W in Eq. (1)) that maximizes classes separability. The separability is measured with the Fisher criterion which is defined as the ratio of the between-class scatter to the within-class scatter and is given in Equation (1).

(1) $$J\left(W\right)=\frac{W^{T}S_{B}W}{W^{T}S_{W}W}$$

where $S_{B}$ and $S_{W}$ are the between classes and total within classes scatters for EEG signal $X\in R^{C\times T}$, respectively, and are calculated as Equations (2), (3), (4), (5).

(2) $$S_{B}=\left(m_{2}-m_{1}\right){\left(m_{2}-m_{1}\right)}^{T}$$

(3) $$m_{1}=\frac{1}{N_{1}}\sum_{n\in C_{1}}X_{n}$$

(4) $$m_{2}=\frac{1}{N_{2}}\sum_{n\in C_{2}}X_{n}$$

(5) $$S_{W}=\sum_{n\in C_{1}}\left(X_{n}-m_{1}\right){\left(X_{n}-m_{1}\right)}^{T}+\sum_{n\in C_{2}}\left(X_{n}-m_{2}\right){\left(X_{n}-m_{2}\right)}^{T}$$

where $C_{i}$ (i = 1 and 2) is the number of i th class samples (in this work, there are P300 and non-P300 classes), and $m_{i}$ (i = 1 and 2) is the mean of samples in i th class. By ignoring the $S_{W}$ matrix, the performance of the algorithm doesn't change significantly because its discrimination effect is smaller than $S_{B}$. In the current study, we propose a new cost function based on the FLD by adding two parameters to increase the effect of $S_{W}$ and $S_{B}$. The linear feature learning (LFL) is calculated as follows:

(6) $$LFL=\max\frac{W^{T}\;\left(S_{B_{N}}-{\beta S}_{W}\right)W}{W^{T}S_{W}W}$$

where in Equation (6) $S_{B_{N}}=S_{b1}+{\alpha S}_{b2}$, $S_{bi}=\left(m_{i}-m\right){\left(m_{i}-m\right)}^{T},i=1,2\text{,}$ and m is the mean of all samples that contain both classes. α and β are hyper-parameters and are calculated optimally based on the grid search for the hybrid RSVP-SSVEP dataset and cross-validation methods for the BCI competitions dataset. The new LFL in Equation (6) improves the separation of the two classes and works better than FLD. To make a balance between the effects of $S_{B}$ and $S_{W}$, the $S_{W}$ matrix is added to the numerator of Equation (6). Moreover, due to the more effect of the larger class $S_{B}$, the hyper-parameter α is added to a class of $S_{B}$. The following eigenvalue decomposition in Equation (7) is utilized to solve the projection matrix W:

(7) $$\left(S_{B_{N}}-{\beta S}_{W}\right)W=\lambda S_{W}W$$

Since P300-based ERP signals have vital information in both spatial and temporal domains, in order to extract features in both domains, the two spatial and temporal projection weights [33] are embedded in Equation (6) and the modified LFL is called spatial-temporal LFL (STLFL). As can be seen in Fig. 2, the main idea is based on two projection matrices $W\in R^{C\times h}$ and $V\in R^{T\times h}$ in two dimensions of spatial and temporal, respectively which map EEG signals $\left(X_{i}\in R^{C\times T}\right)$ to a newly selected features space $\left(f_{i}\in R^{h*h\times 1}\right)$. The parameters *C* and *T* are the number of channels and temporal samples, respectively and *h* is the number of eigenvectors for projection matrix that has a lower dimension than *C* and *T*, in this study. The matrices *W* and *V* are optimized by an iterative algorithm using Equation (8) and Equation (9), respectively as follows:

(8) $$\max\;\frac{\left(W^{T}\left\langle \left(S_{B_{N}}-{\beta S}_{W}\right)VV^{T}{\left(S_{B_{N}}-{\beta S}_{W}\right)}^{T}\right\rangle W\right)}{\left(W^{T}\left\langle S_{w}VV^{T}\;{S_{w}}^{T}\right\rangle W\right)}$$

(9) $$\max\;\frac{\left(V^{T}\left\langle \left(S_{B_{N}}-{\beta S}_{W}\right)WW^{T}{\left(S_{B_{N}}-{\beta S}_{W}\right)}^{T}\right\rangle V\right)}{\left(V\left\langle S_{w}WW^{T}{S_{w}}^{T}\right\rangle V\right)}$$

where ⟨.⟩ denotes ensemble averaging operator over the epochs under the same condition. With the initial identity matrix for V the optimal weights of W are calculated using Equation (8) and are used to calculate the optimal weights of V in Equation (9). The algorithm stops when the error criterions ${\left\|W\left(n\right)-W\left(n-1\right)\right\|}_{2}$ and ${\left\|V\left(n\right)-V\left(n-1\right)\right\|}_{2}$ decreased to a value of $10^{-5}$ where ${\left\|.\right\|}_{2}$ denotes norm 2. Finally, by computing the optimal parameters W and V the new features $f_{i}=vec\left({W^{T}\times X}_{i}\times V\right),i=1,2,\ldots N$ are obtained where vec(.) denotes vectorization. The STLFL code description is given in Algorithm 1.

Algorithm 1 **STLFL**

• **Hyper-parameters optimization**

For the small number of the dataset (hybrid RSVP-SSVEP), the hyper-parameters were selected using the grid search method. The grid search algorithm trains a model on the training set for each combination of hyper-parameters and evaluates its performance using a validation set. After evaluating all combinations, the algorithm identifies the combination with the best accuracy and uses it to train the final model, which is then tested on the test set of data.

However, for the larger number of datasets (BCI competitions dataset), a cross-validation approach was used to determine the optimal hyper-parameters of the model. Cross-validation is more robust, less prone to overfitting and bias, and more computationally efficient compared to grid search, especially with large datasets. To find the best hyper-parameters the method evaluates the model on multiple different folds of the training data with the following steps:

1. *Split the training data into k folds*: The training data is randomly divided into *k* equal folds.
2. *Train the model on k ‒ 1 folds*: For each iteration of cross-validation, *k* ‒ 1 folds are used to train the model.
3. *Evaluate the model on the remaining fold*: The model is then evaluated on the remaining fold, which serves as the validation set for that iteration.
4. *Repeat steps 2 and 3 for k iterations*: This process is repeated *k* times, with each fold serving as the validation set once.
5. *Average the results*: The results from all *k* iterations are aggregated and averaged to obtain an estimate of the model's performance.
6. *Select the best hyper-parameters*: Steps 1–5 are repeated for different combinations of the hyper-parameters. The hyper-parameters that result in the highest average performance are selected as the best hyper-parameters for the model.
7. *Train the final model*: The final model is trained using the entire training data with the optimal hyper-parameters selected in step 6.
8. *Test the model*: The trained model is then tested on the test data to evaluate its performance on new, unseen data.

In the current paper, the parameter *k* is 5 and 3 for BCI competitions III dataset II and II dataset II, respectively.

#### 2.2.2 Discriminative restricted Boltzmann machine (DRBM) classifier

Since the P300 signals are subject-dependent, so powerful classification technique such as DRBM is required to detect P300 from non-P300 signals [36,37,48]. The DRBM is a discriminative form of the RBM, meaning that it is specifically designed for supervised learning tasks. It consists of three layers of neurons: visible units, hidden units, and output units. Furthermore, in the structure of RBM, there are no links between the neurons of the same layer (i.e., restricted), and this causes faster training of the network which is crucial for real-time implementation in BCI applications. In contrast, DRBM models the joint distribution of inputs and target classes, which results in better classification performance in comparison with traditional neural networks.

This study utilized a hybrid DRBM for its classification approach that combines the strengths of both discriminative and generative modeling techniques. In traditional DRBM, the model is trained in a discriminative manner, where it learns to differentiate between P300 and non-P300 classes. However, hybrid DRBM adds a generative component to the model, which allows it to also generate new samples from the learned distribution. During training, the model learns to reconstruct the input data from the visible layer and to differentiate between the P300 and non-P300 classes from the hidden layer. The generative component of the hybrid DRBM allows it to generate new samples that resemble the original input data, even if they do not perfectly match the training data. This feature is particularly useful in situations where there is a limited amount of training data available or when the distribution of the input data is complex and hard to model accurately using only a discriminative approach. More detail about the implementation formula is provided in Supplementary Material.

As previously explained, the P300 classification is divided into two classes P300 and non-P300. However, to detect the target character (in the hybrid dataset, a group selection between nine groups, and in the BCI competitions dataset, a choice of one of six rows and one of six columns), it is necessary to average the P300 scores corresponding to each character on the total repetitions, and finally, the maximum score corresponding to the character is determined as the selected character. It is calculated for j characters and m repetitions of score S as Equation (10) and target character is given by Equation (11).

(10) $$C\left(j\right)=\sum_{k=1}^{m}S_{j}\left(k\right)$$

(11) $$Target\;character={\arg\;\;\max}_{1⩽j⩽J}\;C\left(j\right)$$

## 3 Simulation results

To evaluate the performance of the P300, proposed algorithm applied to the BCI competitions dataset, the results are compared with the results of the previous works focusing on the STDA [35], SVM [49], CNN [40], event-related potential net (ERP-NET) [37], parallel spatial-temporal DRBM (PST-DRBM) [36], DRBM [42], LDA, locality sensitive discriminant Analysis (LSDA) [38], maximum margin criterion (MMC) [24], and group-sparse Bayesian linear discriminant analysis (gsBLDA) [29] techniques. Moreover, the performance of the proposed algorithm is evaluated using an RSVP signal from a hybrid RSVP-SSVEP dataset and the results are compared with the previous works [45] (only RSVP results of the literature [45]), the results obtained using the STDA method [35], LSDA [38], MMC [24]. The range of parameters α and β is set to [1:0.2:5] and [0,0.01,0.001,0.0001,0.1,0.3,0.5,0.7,1,1.5,2], respectively and the dimension reduction variable of STLFL (i.e., h) is set to 4. The maximum iteration of n=500 was required to stop the STLFL algorithm. To validate the performance of the proposed algorithm, we applied 3-fold cross-validation for the hybrid dataset (contains 72 spelling characters) according to Ref. [45] and 15% of data were used for the validation set.

### 3.1 Hybrid dataset results

**Fig. 3:**
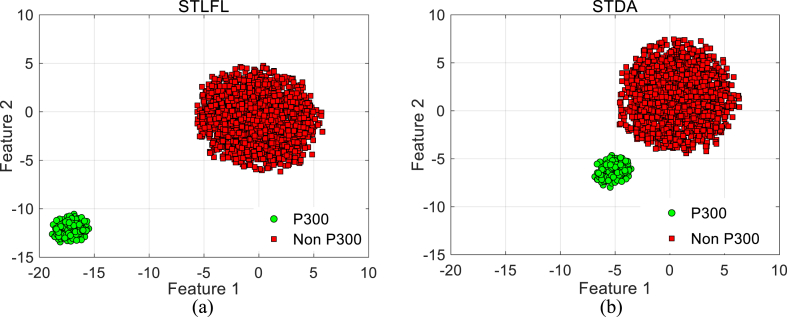
The *t*-SNE visualization of feature distribution for P300 and non-P300 classes in subject “4” from the RSVP hybrid dataset. (a) STLFL features. (b) STDA features [35].

Fig. 3 shows the typical distribution of two of the most discriminative features extracted by the proposed STLFL technique (Fig. 3(a)) and the STDA method (Fig. 3(b)) [35] using t-distributed stochastic neighbor embedding (*t*-SNE) visualization technique for subject “4” from the P300 analysis of the hybrid dataset. To measure the separability of visualization discriminative features, the pointwise biserial correlation coefficient is defined as Equation (12) [50]:

(12) $$r=\frac{\sqrt{N_{1}N_{2}}}{N_{1}+N_{2}}\frac{mean\left\{f_{i}|l_{i}=1\right\}-mean\left\{f_{i}|l_{i}=2\right\}}{std\left\{f_{i}|l_{i}=1,2\right\}},i=1,\ldots ,N$$

where $N_{1}$ and $N_{2}$ are the numbers of P300 and non-P300 samples respectively, and $f_{i}$ and $l_{i}$ are the feature and class label of the *i*-th sample, respectively. In the current study, the square of r ($r^{2}$-value) is used to indicate the higher discriminatively of the features. The larger between-class scatter and smaller within-class scatter are obtained using the proposed STLFL method. The *r*^2^-value of feature 1 and feature 2 are 0.83 (0.4 for the STDA algorithm) and 0.71 (0.48 for the STDA algorithm) for the STLFL algorithm, respectively. Moreover, the p-value between feature 1 and feature 2 for target and non-target classes were applied which achieved 0.004 and 0.0002 for the STLFL features and 0.004 and 0.77 for the STDA features, respectively. Table 1 summarizes the average classification accuracy of the proposed DRBM classifier over six subjects for the group selection using STLFL features in comparison with the RSVP accuracy reported in the literature [45] and the results obtained using the STDA method [35] for the RSVP hybrid dataset. The Wilcoxon signed-rank test was used to test the statistical difference between the proposed method and the STDA. The results clearly show the high accuracy of the classification using the proposed method in all repetitions (p < 0.05 in repetitions 1–5 repetitions).

**Table 1:** The average of group selection accuracy (± one standard deviation) over six subjects using the proposed algorithm (STLFL is used as the feature extraction method and DRBM for classification) for the RSVP hybrid dataset in comparison with the RSVP accuracy of [45], the results obtained using STDA + LDA method [35], RAW + LDA, MMC + LDA [24], and LSDA + LDA [38].

Number of repetitions	Average of RSVP accuracy (%)						*p*-value (STDA [35] vs. STLFL + DRBM)
	[45]	RAW + LDA	MMC + LDA [24]	LSDA + LDA [38]	STDA + LDA [35]	STLFL + DRBM	
1	59.9	32.6 ± 7.8	50.6 ± 10.9	39.1 ± 8.8	49.1 ± 6	67.5 ± 4	0.0021
2	77.1	33.9 ± 5.6	67.08 ± 12.1	41.3 ± 5.7	73.8 ± 5	84.2 ± 2.5	2.2123e-04
3	86.1	37.0 ± 8.6	80.4 ± 5.9	37.8 ± 6.3	90.3 ± 3	93.5 ± 1	0.0494
4	90.5	50.4 ± 9.9	85.8 ± 6.4	48.9 ± 6.5	92.4 ± 4	96.3 ± 1	0.0251
5	96.3	62.9 ± 7.2	91.0 ± 4.1	68.15 ± 9.5	96.7 ± 1.4	98.4 ± 0.5	0.0245

Note: *p* < 0.05 and RAW indicates the filtered EEG signal without any feature extraction.

### 3.2 BCI competitions results

**Fig. 4:**
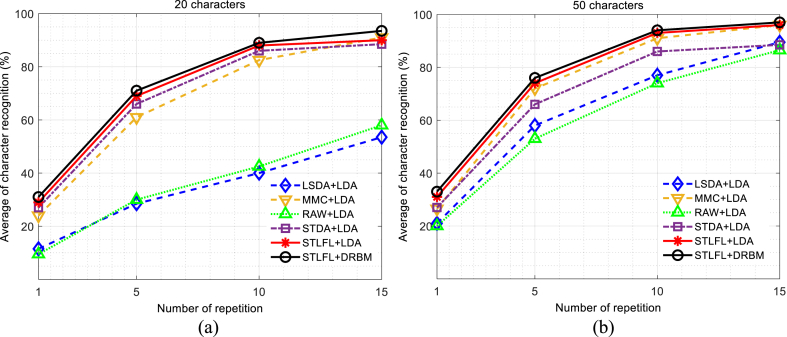
Average character recognition accuracy between two subjects “A” and “B” using proposed STLFL + LDA in comparison with some methods based on the linear discriminative feature extraction methods fed to the LDA classifier in BCI competition III dataset II. (a) For 20 training characters and (b) for 50 training characters.

In this section for further validation, we evaluated the results of the proposed P300 method applied to BCI competition III dataset II and BCI competition II dataset II and the results are compared with the previous authentic works in the state-of-the-art dataset. The motivation for the development of the proposed method in this paper is the achievement of suitable performance with the small number of train samples in the real-time implementation of BCIs. Therefore, to investigate the performance of the proposed method under the small number of train samples, we applied 20 and 50 characters of training samples (there are 85 characters entire training dataset) for learning the model to evaluate the 100 test characters. For each character, there are 180-time samples. This analysis enabled us to assess the performance of the STLFL method using a limited number of training samples. Fig. 4 shows the typical accuracy of character recognition for the proposed STLFL + LDA and STLFL + DRBM method (STLFL is used as the feature extraction method and LDA and DRBM for classification) for the average between two subjects “A” and “B” in comparison with the LSDA + LDA (LSDA is used as the feature extraction method and LDA for classification), STDA + LDA algorithm [35], MMC + LDA, and RAW + LDA (The filtered EEG signal fed to the LDA). The results of Fig. 4(a) and (b) for 20 and 50 characters, respectively indicate that although the small training samples degrade the accuracy of character recognition, the proposed STLFL method after 1, 5, 10, and 15 repetitions could efficiently enhance discriminative ability in comparison with the other methods. The mean accuracies for two subjects with 20 training characters for the proposed STLFL + DRBM method after 1, 5, 10, and 15 repetitions are 31, 71, 89, and 93.5%, and for the proposed STLFL + LDA method after 1, 5, 10, and 15 repetitions are 29, 69, 88, and 90%. The mean accuracies for the STDA + LDA method are 27, 66, 86, 88.5%, for the LSDA + LDA method are 11.5, 28.5, 40, 53.5%, for the MMC method are 24, 61, 82.5, 91%, and for RAW + LDA 9.5, 30, 42.5, 58%, respectively.

**Fig. 5:**
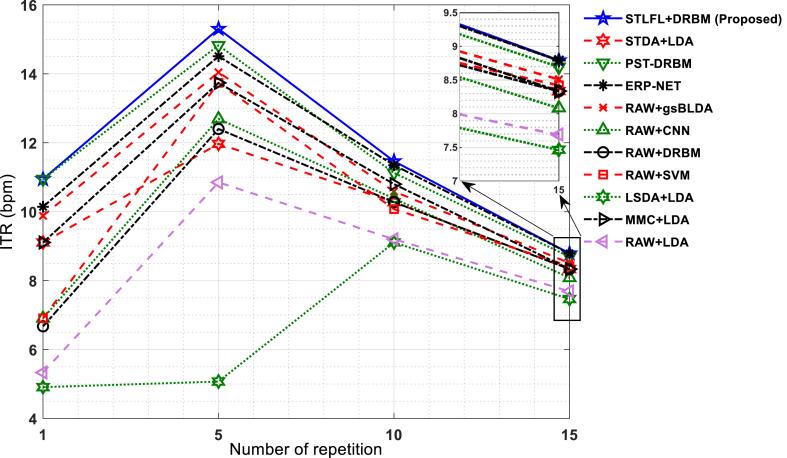
ITR comparisons between STLFL + DRBM and other methods based on the average accuracy of two subjects A and B from BCI competition III dataset II.

Various state-of-the-art methods have been applied to the BCI competitions (III and II) dataset [29,36,37,40,42,49,51] using the entire training dataset to train their models. To enable a fair comparison with these methods and to further assess the effectiveness of the proposed method on a larger dataset, the entire training dataset has been utilized. Table 2 summarized the results of the proposed P300 analysis for character recognition in comparison with the results of the methods STDA + LDA [35], SVM [49], CNN [40], ERP-NET [37], PST-DRBM [36], DRBM [42], LDA, LSDA [38], MMC [24], and gsBLDA [29] in BCI cpmpetition III dataset II. The results show excellent classification performance (average character recognition accuracy of 33.5, 78.5, 93.5, and 98.5% after 1, 5, 10, and 15 repetitions, respectively) in comparison with the results of the previous works. Although the PST-DRBM achieved similar accuracy in higher repetitions, its accuracy was degraded in low repetitions. Furthermore, PST-DRBM has more parameters to be optimized [52] compared to our proposed method, so makes a strong barrier to real-time implementation. The information transfer rate (ITR) in bit per minute (bpm) is applied to demonstrate the character recognition speed of speller as Equation (13):

(13) $$ITR=\frac{\left(\log_{2}\;N+P\;\log_{2}\;P+\left(1-P\right)\log_{2}\frac{1-P}{N-1}\right)}{T}$$

where N and P are the numbers of character present in the speller paradigm (N = 36) and character recognition accuracy, respectively, and T is the time required for character recognition and is calculated as Equation (14):

(14) T=2.5+2.1×rep,1≤rep≤15

where 2.5s is pause between each character epoch and 2.1s is ((100 ms + 75 ms) × 12)/1000. Fig. 5 shows the ITR comparisons among the proposed method and different results presented in Table 2 for the average accuracy of subjects A and B in four repetitions (1, 5, 10, 15). As shown in Fig. 5, the higher ITR of the STLFL + DRBM (10.9, 15.3, 11.5 and 8.8 in four repetitions) is achieved in comparison with the results presented in Refs. [29,[35], [36], [37],^40^,^42^,^49^] for all presented repetitions. This means that speed and character recognition accuracy are optimum in all repetitions, especially, 1 and 5.

**Table 2:** The accuracy of character recognition in two subjects “A” and “B” using the proposed P300 method compared with the different results presented in Refs. [29,[35], [36], [37],^40^,^42^,^49^] for BCI competitions III dataset II.

Subject	Repetitions	Algorithms										
		RAW + SVM [49]	RAW + DRBM [42]	RAW + CNN [40]	RAW + gsBLDA [29]	ERP-NET [37]	PST-DRBM [36]	LSDA + LDA	MMC + LDA	RAW + LDA	STDA + LDA [35]	STLFL + DRBM (proposed)
A	1	16.0	15.0	16.0	20.0	22.0	24.0	9.0	19.0	11.0	19.0	25.0
	5	72.0	62.0	61.0	73.0	75.0	75.0	49.0	64.0	53.0	61.0	73.0
	10	83.0	84.0	86.0	88.0	90.0	90.0	75.0	86.0	79.0	83.0	90.0
	15	97.0	99.0	97.0	99.0	99.0	98.0	87.0	95.0	93.0	95.0	98.0
B	1	35.0	35.0	35.0	43.0	42.0	43.0	33.0	41.0	33.0	41.0	42.0
	5	75.0	76.0	79.0	76.0	77.0	79.0	79.0	83.0	74.0	74.0	84.0
	10	91.0	92.0	91.0	91.0	96.0	94.0	89.0	95.0	86.0	93.0	97.0
	15	96.0	93.0	92.0	95.0	98.0	98.0	94.0	97.0	91.0	97.0	99.0
Mean	1	25.5	25.0	25.5	31.5	32.0	33.5	21.0	30.0	22.0	30.0	33.5
	5	73.5	69.0	70.0	74.5	76.0	77.0	64.0	73.5	63.0	67.5	78.5
	10	87.0	88.0	88.5	89.5	93.0	92.0	82.0	90.5	82.5	88.0	93.5
	15	96.5	96.0	94.0	97.0	98.5	98.0	90.5	96.0	92.0	96.0	98.5

Table 3 demonstrates the application of the proposed STLFL + DRBM to the BCI competition II dataset II for further evaluation. The experimental results indicate that the classification performance of STLFL + DRBM outperforms existing state-of-the-art classifiers [29,46,49]. Notably, the results show that after four repetitions, only the proposed STLFL + DRBM achieved a 100% success rate for classification, as demonstrated in the C experiment.

**Table 3:** The accuracy of character recognition in subject “C” using the proposed P300 method compared with the different results presented in Refs. [29,46,49] for BCI competition II dataset II.

Subject	Repetitions	Algorithms									
		RAW + SVM [49]	RAW + SVNN [46]	RAW + sBLDA [29]	RAW + gsBLDA [29]	RAW + gLASSO [29]	RAW + LASSO [29]	LSDA + LDA	MMC + LDA	RAW + LDA	STLFL + DRBM (proposed)
C	1	64.5	61.3	–	–	–	–	61.3	58.0	54.8	71.3
	2	71.0	71.2	–	–	–	–	41.9	45.1	35.4	77.4
	3	83.9	90.3	–	–	–	–	64.5	67.7	61.2	96.8
	4	96.8	100	–	–	–	–	87.1	77.4	80.6	100
	5	100	100	90.3	93.5	100	96.7	90.3	90.3	87.1	100
	10	100	100	100	100	100	100	100	100	100	100
	15	100	100	100	100	100	100	100	100	100	100

## 4 Discussion and conclusions

The speller application allows neuromuscular patients to communicate with the world without using muscles. In a speller system, patients could take advantage of the Internet, write their thoughts, or call their nurse by connecting the speller response to the speaker. Therefore, speed and accurate recognition of spellers are important issues that require robust algorithms to solve the problem of recognition. In this paper, we proposed a new approach for extracting high-level P300 features, and their effectiveness in target detection was evaluated using two state-of-the-art BCI datasets. From the functional point of view, one important challenge in BCI-speller systems is to achieve high ITR. Some attempts have been obtained in the previous works [29,[35], [36], [37],^40^,^42^,^49^] to increase ITR but a satisfactory result has not still been achieved. The current study tried to develop a method to make a trade-off between the accuracy of recognition and ITR . The results showed that the STLFL technique improved the performance of P300 detection and reduced the number of training samples for achieving suitable classification accuracy. The STDA + LDA technique resulted in lower performance in ITR and character recognition accuracy than STLFL + DRBM (Table 2, and Fig. 5). The ITR of the STDA was 9.10, 11.96, 10.27, and 8.33 bpm for four repetitions 1, 5, 10, and 15, respectively, while the ITR of the STLFL + DRBM is 12.88, 15.30, 11.34, and 8.78 bpm, respectively. In Fig. 4, we applied the LDA classifier to the STLFL method to have an accurate character recognition in comparison with other modified LDA techniques which resulted in an improvement of the discriminative ability of the proposed method. The absence of parameters to optimize in LDA eliminates the challenges in comparing results, ensuring a fair comparison. The RAW+LDA and LSDA + LDA lost the character recognition ability in a small training sample, as can be seen in Fig. 4. Although the performance of MMC+LDA enhanced with the increment of the repetitions for 50 characters, its performance in a small number of training (20 characters) degraded for all repetitions.

One of the main benefits of using DRBMs in ERP signal processing is improved classification accuracy. However, in addition to improved accuracy, DRBMs is also computationally efficient and can be trained on large datasets. This makes them a useful tool for ERP signal processing, where large amounts of data are typically collected and analyzed. Overall, the use of DRBMs in ERP signal processing can provide significant benefits in terms of improved accuracy, computational efficiency, and ability to handle large amounts of data. As shown in Table 2, since all 85 training characters (entire training data) were utilized for model training, the dataset size is substantial, and DRBM has exhibited more robust results compared to other models. Additionally, although DRBM was originally proposed for handling large datasets, the proposed P300 detection method (STLFL + DRBM) outperforms other comparison methods in small datasets, as illustrated in Fig. 4. This success can be attributed to our feature extraction approach, which identifies the most informative features in the data and feeds them as inputs to the DRBM. This helps to reduce overfitting and enhance the model's performance. Thus, STLFL + DRBM proves to be a promising approach for P300 detection and character recognition, suitable for use in both large and small datasets. Furthermore, DRBM is a robust technique for transfer learning because it allows us to use a pre-trained DRBM on a large dataset as a starting point and fine-tune it on a smaller dataset. This enables us to leverage the knowledge learned from the large dataset and improve the performance on the smaller one which we will explore further in our future studies.

This study proposed the P300 detection technique (The STLFL for feature learning and DRBM for classification) to achieve higher performance in comparison with the character recognition accuracy reported in the previous works for all datasets (Table 1 for group selection in the hybrid dataset and Table 2 and Table 3 for character recognition in BCI competitions dataset ). However, the performance of the algorithm was evaluated in the state-of-the-art dataset of the speller without considering the real-time speller application. Future research needs to investigate a real-time implementation of speller-based BCI. Although our proposed P300 method achieved high performance in small training data that is efficient for real-time implementation, it is subject-dependent. Further studies are needed to overcome this issue such as the development of a zero-training transfer-learning approach.

## Author contribution statement

Seyedeh Nadia Aghili: Conceived and designed the experiments; Performed the experiment; Analyzed and interpreted the data; Contributed reagents, materials, analysis tools or data; Wrote the paper. Sepideh Kilani: Performed the experiment; Analyzed and interpreted the data; Contributed reagents, materials, analysis tools or data; Wrote the paper. Rami N Khushaba: Contributed reagents, materials, analysis tools or data. Ehsan Rouhani: Analyzed and interpreted the data; Wrote the paper.

## Funding statement

This research did not receive any specific grant from funding agencies in the public, commercial, or not-for-profit sectors.

## Data availability statement

Data will be made available on request.

## Declaration of competing interest

The authors declare that they have no known competing financial interests or personal relationships that could have appeared to influence the work reported in this paper.
