# Supplementary material for: A spatial-temporal linear feature learning algorithm for P300-based brain-computer interfaces
Source: Heliyon. 2023 Apr 11;9(4):e15380. doi: 10.1016/j.heliyon.2023.e15380 (PMC10126938; doi:10.1016/j.heliyon.2023.e15380)
Supplement: Multimedia component 1 [file mmc1.docx]

**Supplementary Material for “** **A spatial-temporal linear feature learning algorithm for P300-based brain-computer interfaces”**

Seyedeh Nadia Aghili ^a^, Sepideh Kilani ^a^, Rami N Khushaba ^b^, Ehsan Rouhani ^c,*^

^a^ *Department of Electrical and Computer Engineering, Iran University of Science and Technology, Tehran, Iran*

^b^ *Australian Centre for Field Robotics, the University of Sydney, 8 Little Queen Street, Chippendale, NSW 2008, Australia*

^c^ *Department of Electrical and Computer Engineering, Isfahan University of Technology, Isfahan 84156-83111, Iran*

*, Correspondence should be addressed to E.Rouhani. (erouhani@iut.ac.ir)

The Supplementary material includes discriminative restricted Boltzmann machine (DRBM) formula for implementation (section 2.2.2). To implement the DRBM, the energy function for input
$x=(x_{1},\ldots,x_{I})$ and target class $y$ is defined as Equation (S.1):

$E\left( y,x,h \right)=-\text{h}^{\text{T}}\text{W}x-\text{b}^{T}x-\text{c}^{T}\text{h}-\text{d}^{T}\vec{y}- \text{h}^{T}\text{U}\vec{y}$ (S.1)

where the parameters $b,c,$ and $d$ are the biases matrices of the visible, hidden, and the output layers respectively, and $\text{h}\text{ =}(h_{1},\ldots,h_{J})\text{ }$is the hidden neurons. $W$ and $U$ are the connecting weights and
$\vec{y}=(y_{1},\ldots,y_{K})$. According to Equation (S.1), the distribution probabilities to values of $y$, $x$ and $\text{h}$ are defined as Equation (S.2):

$p\left( y,x\text{,h} \right)=\frac{exp(-E\left( y,x,h \right))}{Z}$ (S.2)

where in Equation (S.2) $Z=\sum_{x,y,\text{h}} e^{-E(y,x,h)}$ is the normalization constant. Conditional probabilities of DRBM are defined as Equations (S.3-S.5)

$p\left( x_{i}=1 |\text{h} \right)=sigm(b_{i}+\sum_{j} W_{ij}h_{j})$ (S.3)

$p\left( y_{k}=1 | h \right)= \frac{e^{d_{k}+\sum_{j} U_{jk}h_{j}}}{\sum_{k=1}^{K} e^{d_{k}+\sum_{j} U_{jk^{*}}h_{j}}}$ (S.4)

$p\left( h_{j}=1 |\text{y,x} \right)=sigm(c_{j}+\sum_{k} U_{jk}y_{k}+\sum_{i} W_{ji}x_{i})$ (S.5)

where in Equation (S.3) and Equation (S.5) *sigm* is the logistic sigmoid.

For tuning a generative model on the training set, $\mathcal{D}_{train}=\left\{ \left( x_{q} , y_{q} \right) \right\}$, the loss function is defined as Equation (S.6)

$\mathcal{L}_{gen}\left( \mathcal{D}_{train} \right)=-\sum_{q=1}^{\left| \mathcal{D}_{train} \right|} \log\left( p\left( y_{q},x_{q} \right) \right)$. (S.6)

In a classification problem to obtain an accurate prediction, the value of $p(y|x)$ is optimized directly instead of $p(y,x)$, so Equation (S.6) is rewritten as

$\mathcal{L}_{disc}\left( \mathcal{D}_{train} \right)=-\sum_{q=1}^{\left| \mathcal{D}_{train} \right|} \log\left( p\left( y_{q} | x_{q} \right) \right)$ (S.7)

where the posterior probability $p(y|x)$ in Equation (S.7) is designed as Equation (S.8):

$p\left( y_{k}=1 | x \right)= \frac{e^{d_{k}}\prod_{j=1}^{J} 1+e^{c_{j}+U_{jk}+\sum_{i} W_{ji}X_{i}}}{\sum_{\text{k}=1}^{K} e^{d_{k}}\prod_{j=1}^{J} \left( 1+e^{c_{j}+U_{jk}+\sum_{i} W_{ji}X_{i}} \right)}$. (S.8)

To regularize the discriminative training objective in classification tasks, the generative training objective is added to the discriminative by the following criterion [48] as Equation (S.9):

$\mathcal{L}_{hybrid}\left( \mathcal{D}_{train} \right)=\mathcal{L}_{disc}\left( \mathcal{D}_{train} \right){+\gamma\mathcal{L}}_{gen}\left( \mathcal{D}_{train} \right)$ (S.9)

where the weight $\gamma$ adapts the amount of regularization and can be optimized based on the validation set.
